# Exhaled nitric oxide measurements in the first 2 years of life: methodological issues, clinical and epidemiological applications

**DOI:** 10.1186/1824-7288-35-21

**Published:** 2009-07-20

**Authors:** Carmelo Gabriele, Fernando M de Benedictis, Johan C de Jongste

**Affiliations:** 1 Department of Pediatrics, Salesi Children's Hospital, Azienda Ospedaliero-Universitaria Ospedali Riuniti, Ancona, Italy; 2 Department of Pediatric Respiratory Medicine, Erasmus University Medical Center – Sophia Children's Hospital, Rotterdam, the Netherlands

## Abstract

Fractional exhaled nitric oxide (FeNO) is a useful tool to diagnose and monitor eosinophilic bronchial inflammation in asthmatic children and adults. In children younger than 2 years of age FeNO has been successfully measured both with the tidal breathing and with the single breath techniques. However, there are a number of methodological issues that need to be addressed in order to increase the reproducibility of the FeNO measurements within and between infants. Indeed, a standardized method to measure FeNO in the first 2 years of life would be extremely useful in order to meaningfully interpret FeNO values in this age group. Several factors related to the measurement conditions have been found to influence FeNO, such as expiratory flow, ambient NO and nasal contamination. Furthermore, the exposure to pre- and postnatal risk factors for respiratory morbidity has been shown to influence FeNO values. Therefore, these factors should always be assessed and their association with FeNO values in the specific study population should be evaluated and, eventually, controlled for.

There is evidence consistently suggesting that FeNO is increased in infants with family history of atopy/atopic diseases and in infants with recurrent wheezing. These findings could support the hypothesis that eosinophilic bronchial inflammation is present at an early stage in those infants at increased risk of developing persistent respiratory symptoms and asthma. Furthermore, it has been shown that FeNO measurements could represent a useful tool to assess bronchial inflammation in other airways diseases, such as primary ciliary dyskinesia, bronchopulmonary dysplasia and cystic fibrosis. Further studies are needed in order to improve the reproducibility of the measurements, and large prospective studies are warranted in order to evaluate whether FeNO values measured in the first years of life can predict the future development of asthma or other respiratory diseases.

## Introduction

**Figure 1:**
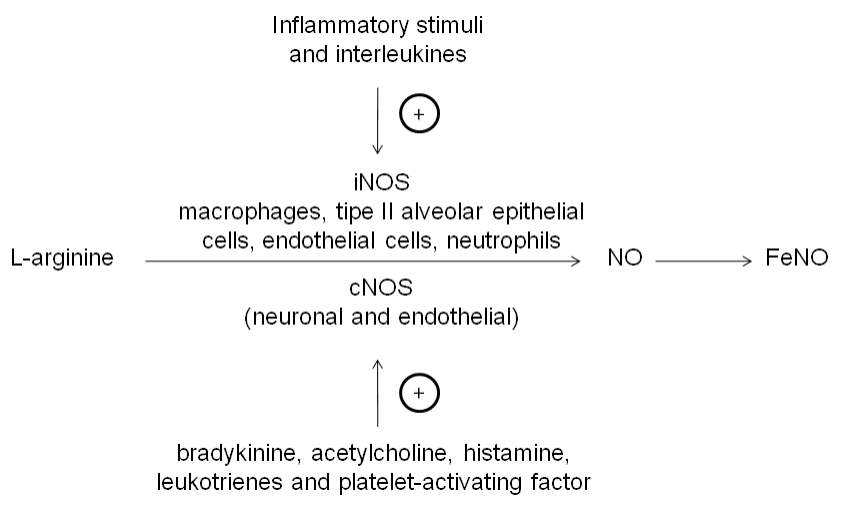
**Synthesis of nitric oxide (NO) from L-arginine**. iNOS: inducible nitric oxide synthases; cNOS: constitutive nitric oxide synthases.

Nitric oxide (NO) is formed in biological systems from L-arginine and oxygen by the enzyme nitric oxide synthases (NOS), of which 3 isoforms have been described: type I and III, which are constitutive (cNOS) and type II which is inducible (iNOS) [1-3] (figure 1). NO is a mediator with a multitude of important regulatory functions and is present in exhaled air in humans [4]. The measurement of nitric oxide in exhaled air (FeNO) is a useful tool to monitor eosinophilic bronchial inflammation in atopic asthma [5]. FeNO measurement has been standardized in cooperative children and adults [6], and normative values have been published for children aged 4 to 17 years [7]. Guidelines for the measurement of FeNO in young children are also available and a number of methodological issues related to the FeNO measurement in infants have been addressed [8]. However, to date there is no standardized technique to measure FeNO in the first 2 years of life.

### FeNO measurement in infants

**Figure 2:**
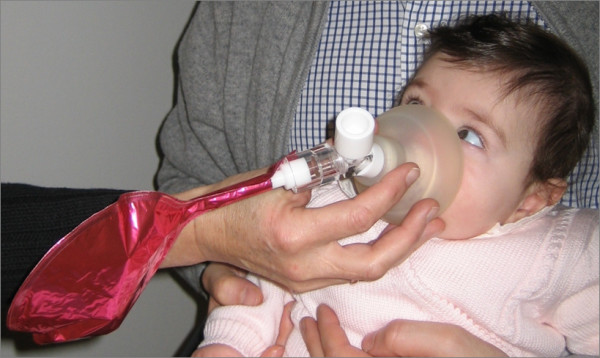
**Off-line FeNO measurement during tidal breathing**. Mixed (oral and nasal) expired air is sampled via a face mask, connected to an NO-inert balloon via a nonrebreathing valve. To reduce contamination by ambient NO, the inspiratory port can be connected to a NO-free air reservoir (not shown). The measurement should be considered successful if the infants maintains a quite tidal breathing during the whole procedure, if the facemask is tightly fitted to nose and mouth and if at least five tidal breaths are collected.

A method appropriate for the measurement of FeNO in infants should be safe, easy to perform and non-invasive. The tidal breathing and the single breath methods have been proposed for the measurement of FeNO in infants and both techniques have showed a good reproducibility and a high success rate [9-12]. The single breath method is often combined with other lung function tests, but requires the sedation of the infant, specialized equipment and well-trained personnel [9,13]. Therefore, it is less suitable for routine testing or large epidemiological studies. The tidal breathing method has been successfully used both on-line (FeNO testing with a real-time display of NO breath profiles) [10] and off-line (collection of exhaled air into receptacles for delayed analysis) [11] (figure 2). This is a simple technique that has the advantages of being non-invasive and can be applied without the use of sedatives [14,15]. However, as FeNO is flow-dependent, with tidal breathing there is a scatter of data depending on the variation in flow rates [6]. The influence of variable breathing pattern on FeNO could be limited by correcting the FeNO values for tidal flow parameters measured during the FeNO sampling [16]. Another option would be the computation of NO output, which takes into account also expiratory flow (NO output = FeNO × tidal flow). However, it has been shown that NO output differentiates children with and without smoke exposure as well as FeNO [10], hinting that the correction for expiratory flow might not be necessary.

The contamination of exhaled air samples with ambient NO has been shown to occur even for low ambient NO concentration, particularly with the tidal-breathing method [15,17]. Hence, it has been suggested that infants always inhale NO free air prior to the FeNO measurement [6,15].

In children and adults the nasal mucosa and the paranasal sinuses are important sources of NO production [6,8]. However, the extent of nasal NO contamination on the orally exhaled NO in infants is unknown. Although oral FeNO values are lower than mixed (oral + nasal) FeNO [11], either nasal or oral NO levels reflect mixed exhaled NO in infants [17]. The use of a two-compartment facemask could reduce nasal contamination, without disturbing the breathing pattern of infants. Nevertheless, the advantages of excluding the nasal contamination during the FeNO measurement in infants have still to be shown.

Spirometric maneuvers have been shown to transiently reduce FeNO levels in asthmatic children [18] and adults [19] and current guidelines recommend to measure FeNO prior to lung function testing [6]. The limited data available in infants have demonstrate no effect of forced expiratory manoeuvres immediately preceding FeNO measurements on FeNO values in sedated infants with airway diseases [11]. Hence, it seems unnecessary to standardize the sequence of these tests.

Several foods and beverages, such as nitrate-reach meals [20] and water consumption [21] have been shown to transiently influence FeNO. In healthy infants it has been show that there is no evidence of an effect on FeNO values of breastfeeding immediately preceding the FeNO measurements [11]. However, such finding should be confirmed also in infants with airways diseases.

Factors not related to the measurement conditions, such as gender, gestational age and anthropometrics have been associated with FeNO also in infants [16,22]. Therefore, these factors should always be assessed and a possible association with FeNO in the specific population under study should be investigated and, eventually, controlled for in multivariable analyses. Increased FeNO values have been found in infants of atopic parents [13]. Also, it has been shown that infants exposed to maternal smoking during pregnancy have lower FeNO than unexposed [10], but this association was subsequently confirmed only in infants of mothers without atopic disease [16]. These findings suggest that maternal atopy modifies the association between FeNO and prenatal smoke exposure. A cross-sectional study by Franklin et al. [23] showed that FeNO was higher in infants exposed to environmental tobacco smoke after birth than in unexposed, suggesting a direct irritant effect of smoke on the infants' airways. However, a prospective birth cohort study recently evaluated the separate effects of pre- and postnatal tobacco smoke exposure in infants. This study showed that infants continuously exposed to smoke both in utero and after birth had lower FeNO than never exposed infants and infants exposed only postnatally, suggesting that the influence of smoking on FeNO depends on the timing and intensity of the exposure [16].

Several methodological issues related to the FeNO measurements in infants have been addressed and further studies in large cohorts of healthy and diseased infants are needed for standardization.

Results of recent studies support the hypothesis that the eosinophilic inflammation seen in asthmatic adults may be present already in wheezy preschool children [24] and in atopic infants [25]. Therefore, the development of a non-invasive bed-side test that could help predicting which infants will develop persistent symptoms would be of great value.

### FeNO and wheezing

Only few studies evaluated FeNO in relation to specific pulmonary diseases early in life and the majority of them were performed with a cross-sectional design in selected populations of infants with a high risk of developing asthma. Baraldi et al. [14] showed that infants with recurrent wheeze had increased FeNO levels during an asthma exacerbation, which rapidly decreased after a course of corticosteroid treatment. Other studies have shown high FeNO levels in infants at increased risk of asthma, such as infants of atopic parents and infants with recurrent episodes of wheezing [13,16,17]. Also, in infants with multiple-trigger wheeze, a reduction of FeNO values has been shown after a course of montelukast or inhaled corticosteroid treatment [26-28]. FeNO has been shown to differentiate infants with various airways diseases already in the first 2 years of life, with the highest levels of FeNO in infants with recurrent wheezing and with atopic predisposition [29]. Such finding was subsequently replicated by Moeller et al. [30] who showed that preschoolers with frequent recurrent wheeze and a stringent index for the prediction of asthma at school age had increased FeNO compared with children with recurrent cough but no history of wheeze. Overall, these findings suggest that the eosinophilic bronchial inflammation seen in asthmatic children and adults could be a characteristic of multiple-trigger wheezy infants, as recently suggested also by a bronchial biopsy study [24]. Hence, FeNO might provide a useful tool to monitor eosinophilic bronchial inflammation early in life. It is noteworthy that previous studies evaluating the association between FeNO and wheezing reached similar conclusions, although the method used to measure FeNO differed between the studies.

Only few prospective studies evaluated the role of FeNO in the development of wheezing and asthma-like symptoms. Latzin et al. [31] showed that a high FeNO after birth was associated with severe respiratory symptoms in the first year of life if the mother had an atopic disease or had been smoking during pregnancy, with the strongest association when both factors were present. These findings would support the hypothesis that only infants who are at higher risk of developing asthma, either due to genetic factors, exposures or both, have higher FeNO. In fact, in the same study a trend towards a negative association between FeNO and severe respiratory symptoms in infants of nonatopic mothers was found [31]. This is in agreement with a recent prospective birth cohort study, which showed that 2 month-old infants with respiratory symptoms had lower FeNO than asymptomatic infants, and that these associations were independent and not modified by maternal atopy [15]. A possible explanation for the negative association between FeNO and respiratory symptoms is that infants participating in these longitudinal studies were not selected because of their health status and therefore did not represent a population of infants at increased risk of developing asthma, who would be more likely to have increased FeNO. Another explanation is that the symptoms assessed in these studies were grouped in one variable, which included not only wheezing, but also other respiratory symptoms that may be due to other mechanisms, including infection. Indeed low FeNO have been found in infants with first time viral-induced acute wheezy bronchitis [32], suggesting that acute viral infection may downregulate NO production [32]. An alternative hypothesis could be that impaired NO diffusion into the airway leading to reduced FeNO might be due to epithelial damage and increased airway secretions in infants with upper respiratory infections [33]. As episodic wheezing associated to viral infections is mostly related to neutrophilic airway inflammation [34], this might explain the reduced FeNO found in infants with viral-induced wheezing.

The limited follow-up of recent prospective studies does not allow at the moment to evaluate whether FeNO measured in the first year of life predicts the development of asthma at school age. Also, the differences in FeNO values reported in previous studies comparing infants with and without symptoms are rather small. Furthermore, it is not possible to evaluate whether the FeNO values obtained in infants correlate with the FeNO measured with a standardized method (on-line or off-line with constant flow) at school age. As children in these cohort studies will grow up, a more clear wheezing pattern will become evident and the diagnosis of asthma will be supported also by the measurement of lung function. Then, it will be possible to evaluate with more precision the predictive role of FeNO measured in infants on the development of asthma at school age.

### FeNO and other pulmonary diseases

FeNO has been explored as a marker of pulmonary diseases that begin early in life, such as primary ciliary dyskinesia, bronchopulmonary dysplasia and cystic fibrosis.

#### Primary ciliary dyskinesia

Primary ciliary dyskinesia (PCD) is an autosomal recessive disease characterized by the lack of effective ciliary motility, causing abnormal mucociliary clearance. This leads to recurrent or persistent respiratory infections in the upper and lower airways [35]. Lower levels of nasal NO have been found in children with PCD as compared to children with bronchiectasis, CF, asthma and healthy controls [36,37]. A recent study has proposed the measurement of nasal NO as a screening test for the diagnosis of PCD, showing that values above a cut-off level of 105 parts per billion could exclude PCD [38]. Children with PCD have low FeNO, but overlap in FeNO values between normal children and those with PCD has been documented [39]. Therefore, FeNO cannot be used to discriminate patients with PCD from healthy subject as clearly as measurements of nasal NO [40]. Larger studies are needed in order to evaluate whether and to what extent FeNO and nasal NO measurements can be helpful for the screening or the diagnosis of PCD in the first years of life.

#### Bronchopulmonary dysplasia and chronic lung disease

Bronchopulmonary dysplasia (BPD) is defined as clinical signs of respiratory distress, chest radiograph abnormalities, and oxygen dependence at 28 days [41]. BPD accounts for the majority of chronic lung disease (CLD) and the airways inflammation of patients with BPD seems to be mostly mediated by neutrophilic granulocytes [42]. It has been shown that school-age BPD survivors have lower FeNO than asthmatics with comparable airflow obstruction, than preterm non-BPD and than healthy children [43]. It has been hypothesized that the pulmonary damage occurring in the early stage of BPD or the reduction of the vascular bed [44] could be responsible of such findings. However, little data is available on the association between FeNO and BPD in the first years of life. Leipala et al. [45] measured FeNO on-line at constant flow in 1-year old infants and showed higher FeNO values in infants with CLD compared to preterm infants without CLD and to infants born at term. Roiha et al. [46] measured on-line tidal FeNO in infants at 1 year and showed that NO output could differentiate between CLD and non-CLD infants better than FeNO. Also, the current authors measured FeNO off-line during tidal breathing in large groups of infants with different airways diseases below the age of 2 years. BPD infants had higher FeNO than CF infants, but lower than atopic infants with recurrent wheezing, taking lung function parameters, tidal volume, and breathing frequency into account [29]. Further studies with longer follow-up are warranted in order to evaluate whether FeNO can be useful to monitor the inflammatory pattern of the airways of BPD infants.

#### Cystic fibrosis

Cystic fibrosis (CF) is a hereditary disease that affects the lungs, digestive system, sweat glands and male reproductive organs. CF lung disease is characterized by chronic neutrophilic airway inflammation, mucus plugging and bronchial infections with specific bacterial pathogens, leading to progressive bronchiectasis and lung damage from which most CF patients die at a median age of 35–40 years. Low FeNO values have been found in both adults and children with CF [47,48] and FeNO has also been shown to correlate with CF severity in children [49]. Although one study previously showed not reduced FeNO levels in CF patients as compared with healthy infants [50], there is a large body of evidence suggesting that infants with CF have lower FeNO values compared with infants with other respiratory diseases and healthy controls [29,51]. It has been hypothesized that the mechanisms underlying the reduced FeNO values in CF patients are related to excess secretions in CF airways that might impair the NO diffusion through the airways [52] or to a primary defect in NO production [53,54]. A recent study by Zetterquist et al. [55] supports the latter hypothesis, by showing that the discrepancy of elevated exhaled NO metabolites and low levels of FeNO are explained by an impaired function or expression of the enzymes involved in the NO metabolism. The differences in FeNO values between CF, healthy infants and infants with other respiratory diseases are relatively small. However, the findings of lower FeNO in CF patients are consistent between studies and the data available seem to suggest that FeNO measurements might be useful in alerting for the diagnosis of CF.

## Conclusion

Practical recommendations for the measurement of FeNO in the first 2 years of life have been published, but to date no standardized technique is available for this age group. Despite different methods to measure FeNO in infants, most findings are comparable among study groups and show consistently high FeNO values in infants at increased risk of developing asthma. Also, specific patterns of FeNO have been shown for groups of infants with different airways diseases. Hence, FeNO might provide a helpful tool for the differential diagnosis of airways diseases in the first years of life. Further follow-up studies in larger groups of infants are needed in order to assess whether and to what extent the measurement of FeNO can predict the subsequent development of airways diseases.

## Competing interests

CG and FMdB declare they have no competing interest.

JCdJ received a research grant from Aerocrine AG, Solna, Sweden (manufacturer of NO analyzers) in 2005 and 2006.

## Authors' contributions

CG drafted the manuscript. FMdB and JCdJ critically revised the manuscript. All authors read and approved the final manuscript.

## Authors' information

CG worked at the Department of Pediatric Respiratory Medicine, Sophia Children's Hospital – Erasmus University Medical Centre (head Prof. JC de Jongste) in Rotterdam, The Netherlands, between 2003 and 2008. During this period he developed several research projects focusing on the measurements of exhaled nitric oxide in infants. This study period will culminate with the dissertation of the PhD thesis to be held at the Erasmus University in 2009. CG currently works as attending physician at the Department of Pediatrics, Salesi Children's Hospital, Azienda Ospedaliero-Universitaria, Ancona, Italy (head Prof. FM de Benedictis).
